# Peripheral vestibular loss in noise-exposed firefighters

**DOI:** 10.3389/fnint.2023.1236661

**Published:** 2023-10-02

**Authors:** Hillary Anne Snapp, Lindsey Vanlooy, Brianna Kuzbyt, Courtney Kolberg, Denise Laffitte-Lopez, Suhrud Rajguru

**Affiliations:** ^1^Department of Otolaryngology, University of Miami, Miami, FL, United States; ^2^Department of Biomedical Engineering, University of Miami, Miami, FL, United States; ^3^Bruce W. Carter Department of Veterans Affairs Medical Center, Miami, FL, United States

**Keywords:** cervical vestibular evoked myogenic potential, cVEMP, noise-induced hearing loss, NIHL, noise-induced vestibular loss, noise, noise overexposure

## Abstract

**Introduction:**

Occupational workers are increasingly aware of the risk of noise overexposure to the auditory system but lack awareness about potential risks to the vestibular system. The purpose of this study was to investigate changes in vestibular end organ function in a known at-risk noise-exposed population, firefighters compared to age- and sex-matched controls using electrophysiologic measures of cervical vestibular evoked myogenic potentials (cVEMP).

**Methods:**

A cross-sectional observational study compared cVEMP response characteristics in 38 noise-exposed firefighters. Firefighters were grouped by years of exposure in the fire service. The cVEMP responses were compared within firefighter groups and between firefighters and age- and sex-matched controls. Dependent variables included the response characteristics of amplitude, latency and threshold.

**Results:**

cVEMP response amplitudes were significantly decreased in firefighters compared to their age- and sex-matched controls. Threshold of the cVEMP responses were significantly higher in firefighters compared to controls and firefighters had a higher incidence of absent cVEMP responses compared to controls. Response amplitudes decreased with increasing years in the fire-service at an increased rate compared to their age- and sex-matched controls. Latency of the cVEMP response was not significantly different in firefighters compared to controls. These findings are consistent with both animal and human studies suggesting noise-induced changes in the sacculocollic pathway.

**Discussion:**

In the absence of any reported vestibular symptoms or auditory indicators of noise-induced hearing loss, these early effects on the vestibular system point to a potential hidden vestibular loss.

## 1. Introduction

Approximately 35% of adults aged 40 years and older experience vestibular dysfunction (Agrawal et al., 2009) the rate of which increases with age (Murdin and Schilder, 2015). Older adults are at significant risk for falls due to imbalance, a leading cause of morbidity and mortality in those over 65 years (Corrales and Bhattacharyya, 2016; Soomar and Dhalla, 2023). Ageing leads to morphological changes in the vestibular periphery and ascending pathways. In the auditory system, noise overexposure has been shown to accelerate degenerative changes to the sensory structures of the inner ear (Kujawa and Liberman, 2006), with concomitant increase in auditory thresholds (Fernandez et al., 2015). Similarly, older adults with noise-induced hearing loss have increased incidence of imbalance and falls (Picard et al., 2008; Girard et al., 2014).

The damaging effect on the inner ear from noise overexposure is linked to both auditory and vestibular system changes. Animal models provide evidence that, similar to hearing loss, vestibular loss is a result of degeneration of hair cells and/or vestibular neurons which are responsible for detection, encoding, and transmission of information about how the body is moving in space. In the vestibular system, the saccule has been shown to be the most susceptible to noise overexposure, and due to its proximity to the stapes footplate the most likely point of injury (Murofushi and Curthoys, 1997; Backous et al., 1999; Stewart et al., 2016). Animal models have demonstrated these effects to be dose-dependent, with broad effects on the saccular structures. High intensity exposures can lead to hair cell loss (Hsu et al., 2008; Tamura et al., 2012), and saccular collapse (Mangabeira-Albernaz et al., 1959). Longer duration of exposure, even at lower intensity levels, can result in macular disruption of the otoliths and hair cell atrophy in noise-exposed rodents (Hsu et al., 2008). These structural changes are reflected in electrophysiologic measures of peripheral vestibular function. Decreased vestibular short latency evoked potential (VsEP) amplitudes and increased latencies in rodents indicate a concomitant functional change occurs in the vestibular system with noise overexposure (Stewart et al., 2020a). However, little is known about the early effects of noise overexposure on the vestibular system. Evidence from auditory studies in animals indicates that overexposure to hazardous noise leads to loss of auditory synapses and spiral ganglion without changes in auditory threshold (Kujawa and Liberman, 2009), yet is associated with delayed noise-induced hearing loss (Fernandez et al., 2015). While there is increasing evidence of the damaging effect of noise overexposure on the vestibular system in animal models, a fundamental gap in translation of these findings to humans is the lack of studies on the effects of chronic or repeated noise exposure over a lifetime. Further, human studies have primarily focused on populations with known noise-induced hearing loss (NIHL) and/or vestibular symptoms (Wu and Young, 2009; Kumar et al., 2010; Akin et al., 2012). To date, there are no prospective studies looking at early vestibular function changes in noise exposed populations without measurable hearing loss and/or vestibular symptoms.

In human studies, much of the work to measure the impact of noise exposure on the vestibular system has focused on cervical vestibular evoked myogenic potentials (cVEMP), an electrophysiologic measure of the sacculo-collic pathway. cVEMPs are short-latency potentials measured from tonically active sternocleidomastoid muscles in response to loud sound, vibration or electrical stimulation of the vestibular system (Welgampola and Colebatch, 2005). The cVEMP is a large potential characterized by a peak at 13 ms (P_13_) and trough at 21 ms (N_21_). Tseng and Young (2013) found that chronic noise exposure leads to abnormal cVEMP responses in 70% of patients. Observed cVEMP abnormalities secondary to noise exposure include absent cVEMP responses, reduced amplitudes, and prolonged latencies (Wu and Young, 2009; Kumar et al., 2010; Stewart et al., 2020b). Research also suggests that the threshold of the cVEMP response is elevated in noise exposed individuals compared those with no history of noise exposure (Akin et al., 2012; Viola et al., 2020).

Furthermore, human studies present evidence of a relationship between cVEMP abnormalities and NIHL. Specifically greater degrees of NIHL have been linked to absent cVEMP response (Akin et al., 2012), prolonged latencies, and reduced amplitudes (Wang and Young, 2007; Kumar et al., 2010). NIHL has also been associated with increased imbalance and higher fall rates (Picard et al., 2008; Girard et al., 2014). Accordingly, occupational groups with known risk for noise overexposure also report high rates of fall-related injuries (Britton et al., 2013; Kim et al., 2018; Phelps et al., 2018; Orr et al., 2019). Despite these associations and the demonstrated risk for saccular damage with noise overexposure, no accepted clinical measure of noise-induced vestibular loss (NIVL) exists. As a test of functional integrity of the saccule and vestibular response pathway, cVEMP shows promising feasibility as a clinical tool to detect noise-induced vestibular changes in humans. However, a major challenge is the paucity of data characterizing cVEMP function in noise-exposed populations. While there is mounting evidence of changes in cVEMP responses in noise-exposed humans, evidence to define the most sensitive parameters to differentiate noise-injury from controls is lacking. Prospective large-scale cross-sectional population studies are needed to further characterize NIVL in humans and to examine the role of the cVEMP response.

Over 22 million occupational workers are exposed to hazardous levels of noise each year (CDC, 2023). Approximately 1 in 8 U.S. workers living with hearing impairment, with occupational exposures accounting for a quarter of the cases (CDC, 2023). As with NIHL, vestibular symptoms are common in occupational workers (Ylikoski et al., 1988; Kilburn et al., 1992) Progressive NIVL increases risk for age-related imbalance, falls, and increased morbidity and mortality (Picard et al., 2008; Girard et al., 2014). Occupational workers are increasingly aware of the risk of noise overexposure to the auditory system but lack awareness about potential risks to the vestibular system (Snapp et al., 2022). Many of the prior studies on NIVL have focused on military personnel, industrial workers, or police officers (Wu and Young, 2009; Kumar et al., 2010; Akin et al., 2012). As highlighted by our recent work, firefighters are amongst those especially vulnerable to NIHL (Williams et al., 2023). This group experiences repeated impulse noise exposure, typically for many years. During emergency responses, firefighters can be exposed to noise levels greater than 115 dBA for upwards of 30 min intervals (Reischl et al., 1981). Common sources of noise exposure for firefighters include truck engines, sirens, alarms, equipment, and tools (Pepe et al., 1985; Ewigman et al., 1990). Although firefighters recognize the importance hearing protection in preventing hearing loss, hearing protection devices are seldom used by this group, as workers feel hearing protection will interfere with their ability to perform critical tasks (Hong et al., 2008). The lack of hearing protection adoption makes this group especially susceptible to noise-induced damage to the inner ear structures. Not only are firefighters exposed to damaging noise levels, but they also have consistent exposure to ototoxins and neurotoxins in smoke. Carbon monoxide and hydrogen cyanide are common ototoxic chemicals encountered by firefighters along with acrolein, benzene, methylene chloride, polyaromatic hydrocarbons, perchloroethylene, carbon monoxide, toluene, trichloroethylene, trichlorophenol, xylene, and formaldehyde (Bolstad-Johnson et al., 2000; Austin et al., 2001; Fechter et al., 2002; Campo et al., 2013).

The purpose of this study was to ascertain changes in vestibular end organ function in a known at-risk noise-exposed population, firefighters compared to age-matched controls using electrophysiologic measures of cVEMP. While the effects of noise exposure on cVEMP have been previously studied in other groups, this has not been examined in a population with repeated noise overexposure. We hypothesized that clinical measures of vestibular evoked myogenic potentials would detect noise-induced changes in the vestibular pathway.

## 2. Materials and methods

### 2.1. Design

This study was designed as a prospective, population-based, cohort study. All study procedures were approved by the University of Miami Institutional Review Board (IRB# 20200222).

### 2.2. Participants

Participants were recruited from South Florida fire stations. To explore the cumulative effects of noise exposure, length of time in the fire service in years was considered as a proxy for noise exposure. Inclusion and exclusion criteria were designed to recruit participants into 3 categories: (1) firefighters with <1 year of service, (2) firefighters with 10–19 years of service, and (3) firefighters with 20^+^ years of service. The first group, with <1 year of service, would have limited exposure to occupational noise. This allowed for comparison with firefighters with longer service and hence cumulative exposure over time (Snapp et al., 2022). Based on our preliminary data, an upper age limit of 54 years was employed to limit age affects. Age- and sex-matched controls were recruited from the general population. Participants and controls were required to be free from service-related exposures and significant recreational exposures for a minimum of 24 h prior to assessment. Participants with a history of head trauma, diabetes, cardiovascular disease, daily tobacco use, pathological dizziness, active otologic pathology, and/or history of ototoxic medication use were excluded.

We limited inclusion of control participants aged 18–45 years to those with audiometrically normal hearing (≤ 25 dB HL) from 500–8,000 Hz. For control participants between 46–54 years, inclusion was limited to those with no more than a mild hearing loss from 3,000–8,000 Hz (≤ 30 dB HL at 3000 Hz, ≤ 35 dB HL at 4000 Hz, and ≤40 dB HL from 6,000–8,000 Hz). Leniency of audiometric thresholds for the high frequencies is consistent with the definition of near-normal hearing outlined previously (Moore et al., 2012; Valderrama et al., 2018).

### 2.3. Procedures

Visual examination of the ear canal and tympanic membrane was conducted to ensure normal anatomy and clear external canals free from occluding debris prior to all assessments. Air-conduction hearing thresholds were measured from 500–16,000 Hz using the Intelligent Hearing Systems (IHS) SmartAUD system (Miami, FL) with ER-2 high-frequency insert earphones.

cVEMP responses were collected and analyzed using the IHS SmartEP system (Miami, FL) under a two-channel electrode montage, with sound delivered through insert earphones. The non-inverting electrode was placed on the high forehead (Cz), the inverting electrode on the ipsilateral sternocleidomastoid muscle, and the common ground electrode on the low forehead (Fpz). cVEMP responses to 500 Hz and 1,000 Hz tone burst stimuli were collected using rarefaction polarity at 120 dB peak SPL, and stimulus presentation order was randomized. Stimuli were presented at a stimulus repetition rate of 5.1/s. At least 2 averaged responses of 80 sweeps were collected to ensure replicability. The positive and negative polarities of biphasic cVEMP waveforms were defined as P_13_ and N_21_ with P_13_ being the first positive peak of VEMP and N_21_ being the following negative trough of the cVEMP waveform. The peak latencies and peak-to-trough amplitudes (P_13_–N_21_) were measured. Two independent judges determined the latencies and peak-to-trough amplitude of the P_13_–N_23_ as well as the threshold level. Interscorer disagreements were resolved with a third reviewer under collective analysis. Participants laid on the testing table in a semi-recumbent position for cVEMP testing. Participants were instructed to lift their head off the table and turn their head away from the stimulated ear. To control for the influence of tonic EMG level on the cVEMP, electromyographic (EMG) and cVEMPs were recorded simultaneously from each side of each subject and EMG amplitude was actively monitored by the tester via the IHS SmartEP to ensure recordings remained within 100–300 μV.

The following response calculations were performed for each participant and compared across groups: (1) P_13_–N_23_ cVEMP amplitudes for 1,000 Hz and 500 Hz, (2) cVEMP thresholds at 500 Hz, (3) P_13_ and N_23_ latencies, and (4) 1000:500 Hz response ratios using P_13_–N_23_ amplitudes obtained at each frequency. To obtain the cVEMP threshold, the sound level was decreased in steps of 5 dB until no response was observed. The lowest sound level at which a cVEMP was present was defined as the threshold.

### 2.4. Statistical methods

Kurtosis and skewness measures were applied to determine if the data were normally distributed. For data that were normally distributed, a one-way ANOVA was used. For data that were not normally distributed, the non-parametric independent-samples Mann–Whitney *U* and Kruskal–Wallis tests were used with Dunn’s correction to account for multiple comparisons. Statistical significance was defined by *p*-values <0.05. Pairwise comparisons with Bonferroni corrections for multiple comparisons were used to follow-up significant main effects. All analyses were performed using SPSS^®^ software (version 28.0; New York: IBM Corp^®^).

## 3. Results

The sample included 38 firefighter participants and 38 age- and sex-matched controls totaling 76 firefighter ears and 76 control ears for study. Participants ranged in age from 20–54 years (mean 36 ± 11 years), years of service ranged from 0–31 years (mean 11 ± 9 years), and 95% of participants identified as male. Participants were classified into three categorical groups: (1) those with less than 1 year in the fire service (28 ears), (2) those with 10–19 years of service (30 ears), and (3) those with 20 years or more of service (18 ears). Demographic details of participants and controls are presented in Table 1. None of the firefighter or control participants reported any perceived changes in balance function, or history of vertigo/dizziness. Both controls and firefighters presented with normal functional mobility as assessed by functional gait and time-up-and-go measures.

**Table 1:** Firefighters vs. controls.

		Sample	Age (years)	Years of service	PTA 2-4 kHz (dB HL)
		(Ears)	Mean, SD	Mean, SD	Mean, SD
<1 year	Firefighter	28	24, 3.6	0.48, 0.45	4.6, 4.6
	Control	28	24, 3.0	NA	6.3, 5.5
10–19 years	Firefighter	30	39, 5.9	14.4, 2.7	10.8, 7.6
	Control	30	39, 5.8	NA	10.5, 7.7
20^+^ years	Firefighter	18	48, 4.2	23.2, 3.4	15.7, 6.5
	Control	18	49, 4.1	NA	13.8, 9.1

**Figure 1:**
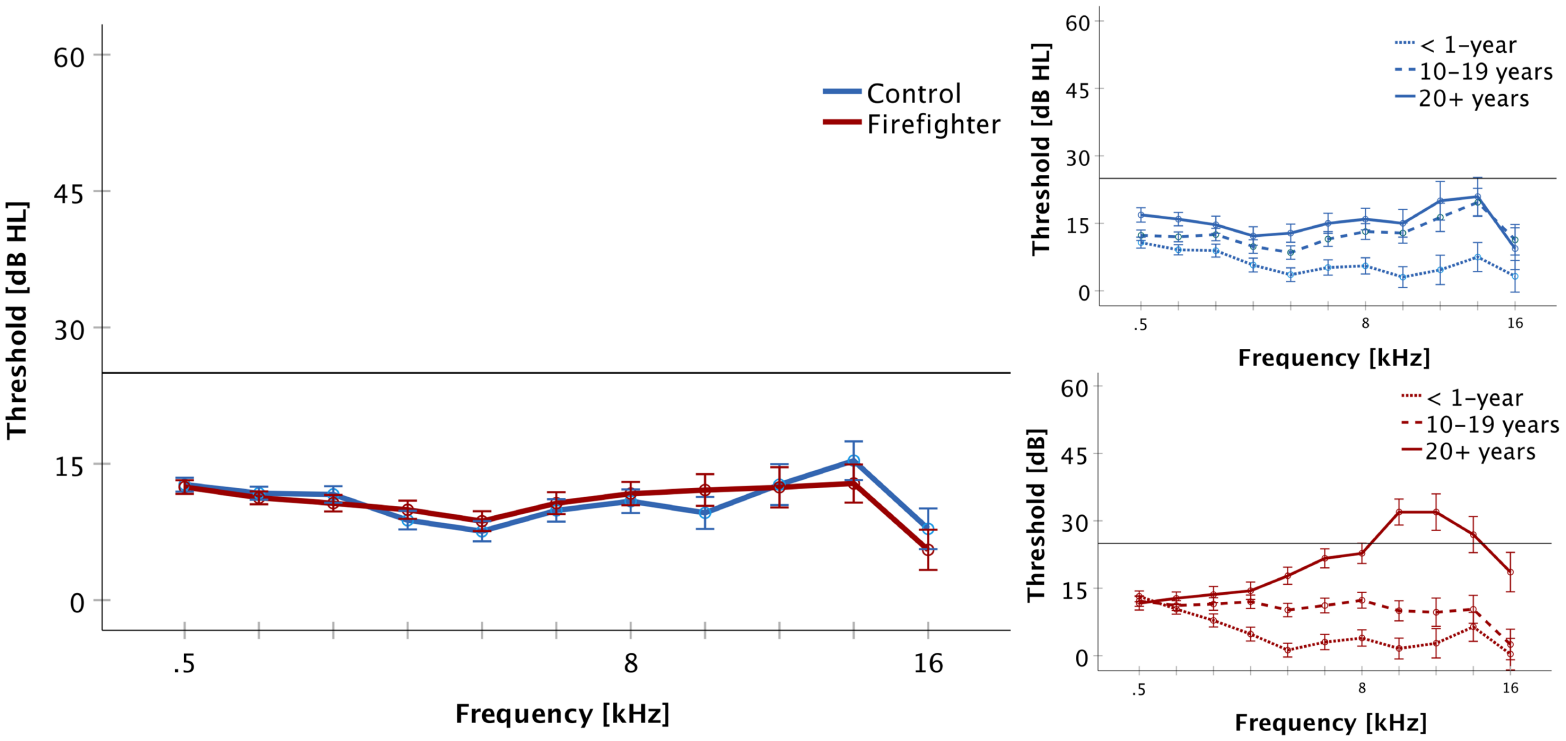
Audiometric thresholds are presented for the frequencies of 500 to 16,000 Hz (Mean + SEM). Firefighters are presented in red and controls in blue. The reference line indicates the limit of normal hearing. There was no significant difference in thresholds between groups and all participants demonstrated normal hearing thresholds (≤ 25 dB HL) for the standard frequencies of 500–8,000 Hz. The right panel presents audiometric results by service group for firefighters and controls. Firefighters with 20^+^ years of service demonstrated increased thresholds in the ultra-high frequency range of 10–16 kHz.

Independent-samples *t*-tests were conducted to assess for audiometric threshold differences between firefighters and controls. There was no significant difference between firefighters and controls for 2–4 kHz or 10–16 kHz pure tone averages. Mean 2–4 kHz PTA was 9.7 ± 7.7 dB for firefighters, and 9.6 ± 7.8 dB for controls. Mean 10–16 kHz PTA was 10.7 ± 17.9 dB for firefighters, and 11.4 ± 15.3 dB for controls. Complete audiometric profiles of participants and controls are presented in Figure 1. One-way analyses of variance were conducted to explore differences at the subgroup level. ANOVA demonstrates significant increase in audiometric thresholds for 10–16 kHz for firefighters with 20^+^ years of service (Mean = 24.4 ± 22.7 dB) when compared to age- and sex-matched controls (Mean = 16.3 ± 13.4 dB; *p* < 0.001), to firefighters with 10–19 years of service (Mean = 8.1 ± 10.5 dB *p* < 0.01), and to firefighters with less than 1 year of service (Mean = 2.8 ± 13.7 dB; *p* < 0.001).

**Figure 2:**
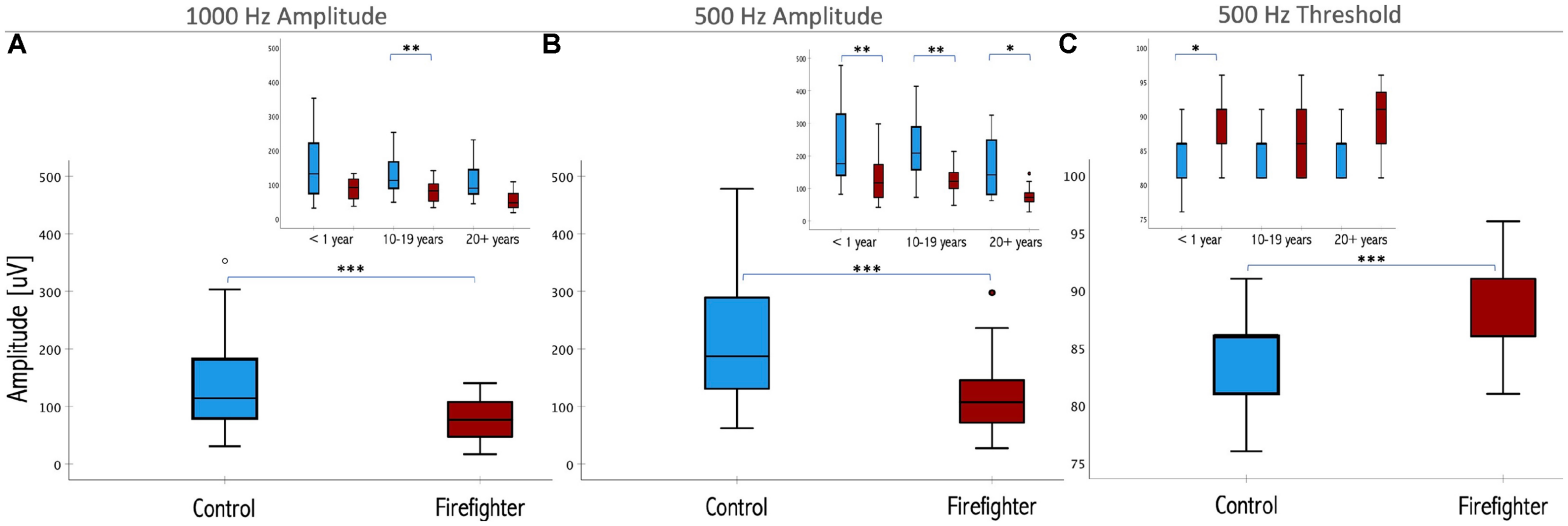
Box plots representing the median and interquartile ranges of P_13_–N_23_ amplitude for **(A)** 1,000 Hz and **(B)** 500 Hz stimuli in firefighters (red) and age- and sex-matched controls (blue). Threshold responses are presented in **(C)**. The error bars above and below the box indicate the 90th and 10th percentiles, respectively. Inset presents the differences by years of service. Significant differences for matched controls are indicated by asterisk (^*^<0.05, ^**^<0.01, ^***^< 0.001; respectively).

Independent-samples Mann–Whitney *U* test was performed to determine if cVEMP responses differed between firefighters and controls. The results indicated that 1,000 Hz amplitudes were significantly reduced in the firefighter cohort compared to those of age and sex-matched controls (*z* = −4.52, *p* < 0.001). The median amplitude response for 1,000 Hz stimuli in controls was 116.8 μV (IQR = 100.5 μV) compared to 73.2 μV (IQR = 61.3 μV) in firefighters (Figure 2A). Independent-samples Kruskal–Wallis test indicated significant differences in cVEMP amplitudes at the subgroup level for 1,000 Hz stimuli (*p* = 0.001). cVEMP amplitudes at 1000 Hz were significantly reduced for firefighters with 10–19 years of service compared to their age and sex-matched controls (*p* < 0.05). Other significant group differences included firefighters with 10–19 years of service to <1 year controls (*p* < 0.05), and firefighters with greater than 20 years of service to the 10–19 years and <1 year control groups (*p* < 0.05; *p* < 0.05, respectively).

**Figure 3:**
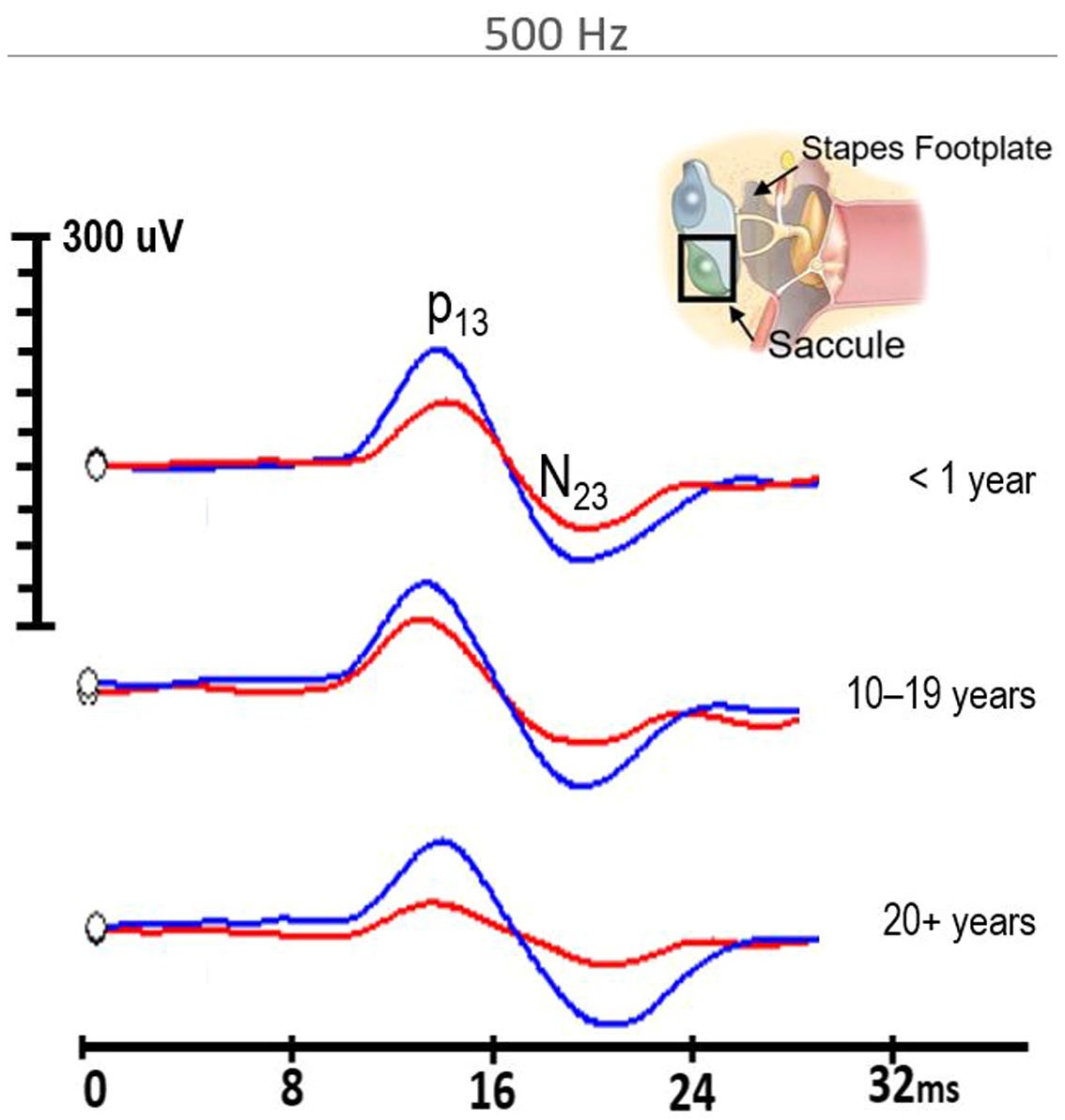
500 Hz cVEMP grand average waveforms for firefighters (red) in the <1 year of service, 10–19 years of service, and 20^+^ years of service groups compared to their age-and sex-matched controls (blue). All firefighter groups demonstrated significantly reduced amplitudes compared to controls (*p* < 0.001).

Similarly, 500 Hz amplitude responses were significantly reduced in the firefighter cohort compared to age and sex-matched controls (*z* = −6.02, *p* < 0.001). The median amplitude response for 500 Hz stimuli in controls was 191.5 μV (IQR = 162.2 μV) compared to 114 μV (IQR = 62.2 μV) in firefighters (Figure 2B). Independent-samples Kruskal–Wallis test indicated significant differences cVEMP amplitudes at the subgroup level for 500 Hz stimuli (*p* < 0.001). cVEMP amplitudes were significantly reduced for firefighters with less than 1 year of service compared to age- and sex-matched controls (*p* < 0.01), firefighters with 10–19 years of service compared to their age- and sex-matched controls (*p* < 0.01), and firefighters with 20^+^ years of service compared to age- and sex-matched controls (*p* < 0.05). Other significant group differences included firefighters with 10–19 years of service to <1 year controls (*p* < 0.01), and firefighters with greater than 20 years of service to the mid- and early-career control groups (*p* < 0.001; *p* < 0.001, respectively). Grand average cVEMP wave forms comparing differences in amplitude responses between firefighters and controls by years of service for the 500 Hz stimuli are presented in Figure 3.

**Figure 4:**
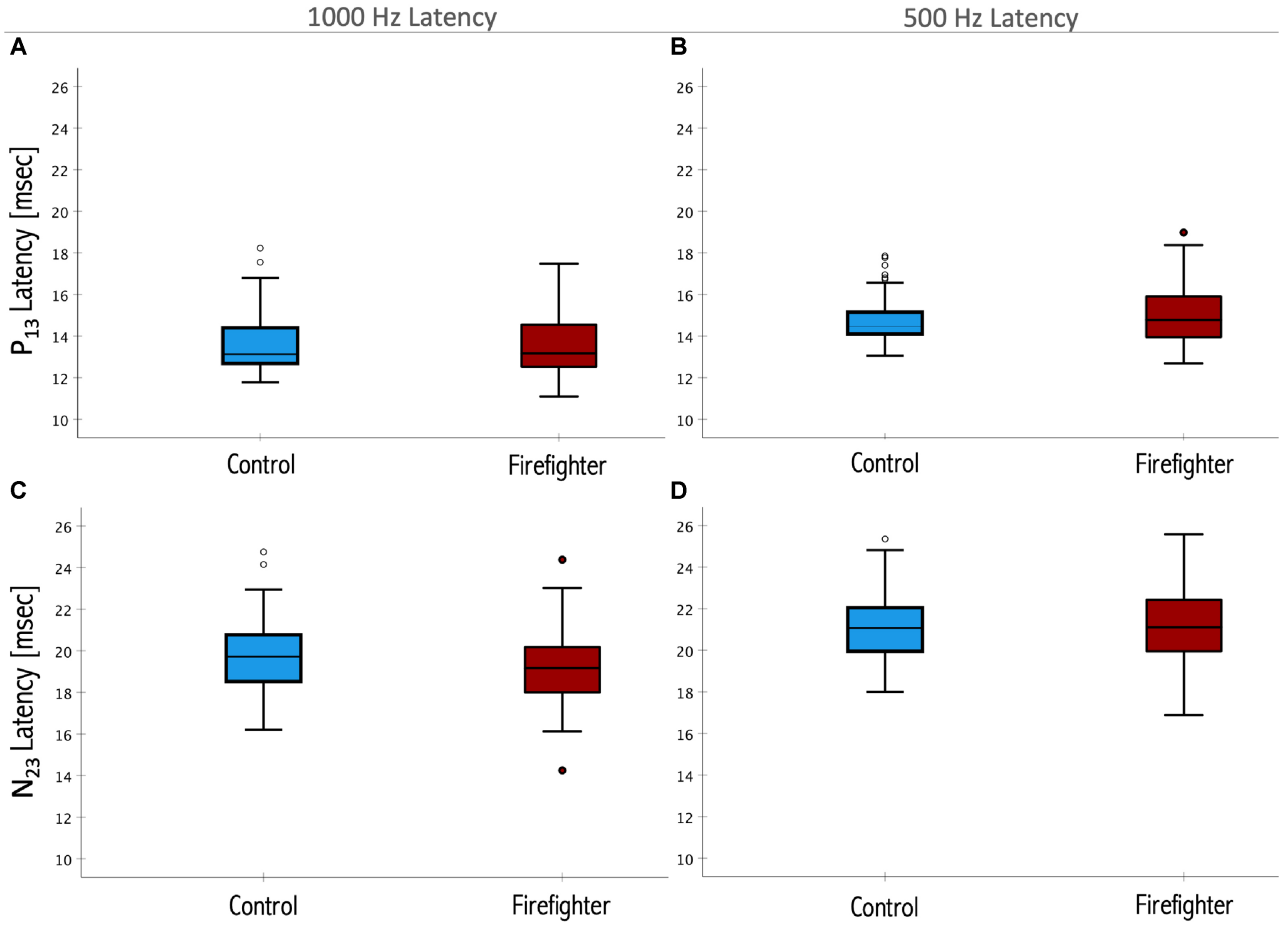
Box plots representing the median and interquartile ranges of P_13_ 1,000 Hz **(A)** and 500 Hz **(B)** latencies and the N_23_ 1,000 Hz **(C)** and 500 Hz **(D)** latencies. Firefighters are presented in red and age- and sex-matched controls in blue. The error bars above and below the box indicate the 90th and 10th percentiles, respectively. Circles indicate outliers. There was no significant difference in the latencies of the response between firefighters and controls for either measure (*p* > 0.05).

There was no significant difference in the 1,000 Hz to 500 Hz cVEMP amplitude ratio between firefighters and their matched controls. There was no significant difference in latency of the P_13_ or N_23_ cVEMP responses between firefighters and their matched controls for either the 1,000 Hz or 500 Hz stimuli (Figure 4).

Figure 2C shows that cVEMP threshold responses to 500 Hz stimuli were significantly higher in the firefighter cohort compared to age and sex-matched controls (*z* = 3.83, *p* = 0.001). After correcting for multiple comparisons, independent-samples Kruskal–Wallis test only identified significance differences for the <1 year subgroup. Although, reliable cVEMP responses were elicited for all control subjects, whereas 15% of the time cVEMPS were absent in firefighters for the 1,000 Hz stimuli. Absent responses were not included in the amplitude or latency analyses.

## 4. Discussion

Studies in both animals and humans have established that noise overexposures can lead to changes in the sensory structures of the cochlea that are not necessarily reflected in functional changes in hearing (Liberman et al., 2016). Yet, noise exposed ears are more likely to experience hearing loss to a greater degree than non-exposed ears with ageing (Kujawa and Liberman, 2006). Given the proximity of the vestibular endorgans, in particular the saccule to the stapes footplate, it follows that concomitant damage to these structures may also occur with noise overexposure. Accordingly, animal studies in noise-exposed ears provide evidence of neuronal and hair cell loss in the vestibular end organs, in particular the calyx endings on afferent neurons of the Type I hair cells (Hsu et al., 2008; Stewart et al., 2020a). Further evidence correlates VsEPs with significant loss of ribbon synapses, which occurs in the absence of hair cell or neuronal loss in the otoliths (Wan et al., 2019), consistent with the degeneration of the auditory synapses that occur in the absence of functional changes in hearing with noise overexposure.

This study aimed to characterize changes in the functional integrity of the saccule and vestibular response pathway of occupationally noise exposed adults using cVEMP. Firefighters were recruited into three groups based on years of occupational exposure to explore cumulative effects of noise. Results were compared to age- and sex-matched controls. As we have previously shown (Snapp et al., 2022), audiometric thresholds for the standard frequencies of 0.5–8 kHz are largely normal in firefighters while measures in the extended high frequency range of 10–16 kHz reveal significantly higher thresholds in firefighters with 20^+^ years in the fire service compared to their age- and sex-matched controls. These findings are consistent with those of others (Skerkova et al., 2021) demonstrating that changes in high frequency thresholds may be an early indicator of noise induced damage to the inner ear.

Despite otherwise largely unremarkable audiometric findings and no reports of imbalance or dizziness, firefighters showed significant reductions in cVEMP amplitudes for both 1,000 and 500 Hz stimuli compared to age- and sex-matched controls. Larger effects are observed for the 500 Hz stimuli, suggesting this stimulus frequency may be more sensitive to detecting changes in the sacculocollic pathway associated with noise overexposure. Comparisons by years in the fire service demonstrate significant reductions in amplitudes even in the group with less than 1 year of exposure, suggesting the effects of noise overexposure on the vestibular system occur early. The cumulative effects of exposure over time are also observable in the data, with cVEMP amplitudes decreasing for both 1,000 Hz and 500 Hz stimuli with increasing years in the fire service. Review of the grand average waveforms in Figure 3 show the clear morphological changes in the firefighters cVEMP responses with increasing exposure, while controls maintain robust and easily identifiable cVEMP responses that do not significantly change overtime. cVEMP amplitude is an indicator of changes in the sacculocollic pathway (Baier et al., 2009; Taylor et al., 2012). Reduction in the cVEMP amplitude of firefighters compared to age and sex-matched controls is indicative of reduced saccular function that outpaces that which is observed by ageing alone. VEMP responses are known to be affected by ageing (Layman et al., 2015; Li et al., 2015), although control participants in this sample do not demonstrate significant changes in amplitude of the cVEMP response. This could be explained by the participant demographics, as all were under 55 years of age. Other investigations into the effects of age on cVEMP have found there is an age-related decrease in cVEMP amplitude in the absence of known pathology, but significant changes are limited to older adults (Brantberg et al., 2007; Rosengren et al., 2011; Agrawal et al., 2012), with no significant differences in the cVEMP response in those under 50 years of age (Singh et al., 2014). The median amplitude responses over time for the control participants (Figure 2, insets), do present a trend of decreasing response amplitude, suggesting an effect of age on cVEMP amplitudes. However, the amplitude changes are accelerated in firefighters for both 500 and 1,000 Hz stimuli, signaling an exposure-age interaction similar to that which has been shown in noise-exposed auditory systems (Kujawa and Liberman, 2006).

The 1,000 to 500 Hz amplitude ratio provides insights into the tuning of the cVEMP response. It has been shown that tuning varies with age (Piker et al., 2013), and is altered in pathologic ears (Rauch et al., 2004; Angeli and Goncalves, 2019). This has largely been attributed to the mechanical properties of the otoliths, where altered tuning is thought to reflect changes in the mechanical resonance of the saccule (Rauch et al., 2004). Exploration of cVEMP tuning in noise-exposed firefighters using the 1,000 and 500 Hz amplitude responses was unremarkable. Specifically, tuning was not significantly different in firefighters compared to age- and sex-matched controls. Firefighters in our cohort did not report vestibular symptoms, particularly as they’d relate to BPPV, that would be indicative of changes in the mechanical tuning. Previous research indicates that vestibular noise-injury is selective for Type I hair cells affecting the synaptic process (Hsu et al., 2008; Stewart et al., 2020a) which alone would not necessarily be expected to affect the mechanical resonance of the sacculus. According to the accelerometer-seismometer model of otolith function, at low frequencies the otoconia move relative to the macula whereas at high frequencies the otoconia remain stationary while the macula moves (Piker et al., 2013; Grant and Curthoys, 2017). It is possible that higher frequency responses may be more sensitive to changes in the membrane. For this reason, we have also established an animal model to characterize the tuning of the cVEMP response [see accompanying paper (Raciti et al., 2023)].

There was no significant difference in latency of the P_13_ or N_23_ response between firefighters and controls. The response latencies reflect the neural conduction time. Our results suggest that the neural conduction of the response along the sacculocollic pathway is not affected in the exposed group when compared to controls. Other studies have reported that cVEMP latencies are largely unaffected in young adults (Rosengren et al., 2011; Layman et al., 2015; Li et al., 2015). In combination with our findings, this suggests that the latency parameter of the cVEMP is likely insensitive to noise induced changes to the saccule in human subjects. The properties of the cVEMP response in noise-exposed firefighters, specifically significant reductions in amplitude while latency remains unaffected, reflect changes occurring at the level of the vestibular receptors.

cVEMP threshold responses in noise-exposed firefighters are elevated compared to their age-and sex-matched controls. While cVEMP thresholds have been found to be elevated in populations with NIHL (Wang and Young, 2007; Wu and Young, 2009; Akin et al., 2012), our findings are the first in noise-exposed ears with normal hearing. Animal studies of cochlear synaptopathy show ABR thresholds recover after subthreshold noise injury (Kujawa and Liberman, 2009). Although, this pattern is in response to a single controlled noise overexposure with thresholds recovering over several weeks. Our findings suggest thresholds remain elevated in firefighters compared to controls which may be related to the chronic repeated noise overexposure experienced in this population. We also found higher rates of absent cVEMP responses in the exposed group compared to controls. Studies exploring the effects of ageing on the cVEMP response indicate that cVEMPs are consistently present in young adults, and that age-related absent responses increase in frequency after 50 years of age (Janky and Shepard, 2009). Given our comparatively young cohort of participants (i.e., <55 years), this is consistent with our findings of present cVEMP responses in all control ears. Conversely, cVEMPs were absent in 15% of firefighter ears. Absent responses almost entirely occurred in those with 20^+^ years of service, indicating that there may be cumulative risk of noise-induced vestibular loss over time. This is consistent with studies in populations with established NIHL highlighting the relationship of increasing NIHL to loss of cVEMP responses (Wang and Young, 2007, Wu and Young, 2009, Akin et al., 2012).

There is mounting evidence in both human and animal models that noise overexposure negatively affects the vestibular system. Several human studies have shown changes in the cVEMP response in individuals with NIHL (Wang and Young, 2007; Akin et al., 2012; Tseng and Young, 2013). Less is known about the effects of noise on the human vestibular system in noise-exposed populations without hearing loss. Moreover, we do not understand enough about the critical limits of noise exposure on the vestibular system or the progressive profile of noise-induced vestibular loss, leaving critical gaps for early identification and prevention. However, the robust evidence of the effects of noise on the auditory system would suggest that a similar characterization may be seen in the vestibular counterpart. Wherein noise effects will demonstrate cumulative effects on the vestibular system that are delayed in presentation relative to the exposures, leading to an acceleration in age-related vestibular loss. Toward this, we explored the effects of noise on the cVEMP response and found significant differences in saccular function in a high-risk, chronically noise-exposed population. The pattern of changes in the amplitude of the cVEMP response in the firefighter group indicates an interaction of ageing and exposures that suggest early ageing. Review of the data here indicates a progressive decrease in amplitude of the response that outpaces age-matched controls. Our findings provide evidence of early changes to the sacculocollic pathway starting within the first year of work in the fire service, even in the absence of measurable changes in hearing function. This is the first study to explore cVEMP responses in occupationally noise-exposed group with essentially normal hearing thresholds. This is also the first study to explore variation in vestibular responses by years of exposure and provides evidence of the cumulative effects of noise overexposure on vestibular function.

Indeed, human studies come with challenges, and many factors cannot be fully controlled. Unlike in preclinical studies, it cannot be assumed that a single subject experiences equal exposure to both ears. There is increasing evidence of asymmetric NIHL (Segal et al., 2007; Akin et al., 2012; Sturman et al., 2018; Aarhus and Engdahl, 2020; Sommerfeldt et al., 2021) and NIVL profiles (Wang and Young, 2007; Akin et al., 2012). In occupational workers in particular, shadowing from the head positioning during tasks and other environmental factors may lead to differences in exposures at each ear [summarized in Le et al. (2017)]. Another challenge is the large inter-subject variability in the cVEMP amplitude response due to difficulty controlling the SCM activation, variations in test methods, reliance on surface electrodes, etc., McCaslin et al. (2013) which all serve to limit its diagnostic utility. However, far less variability is observed in the data of the exposed group compared to controls. Review of the data in Figure 2 suggests that the exposed group does not achieve as large of amplitude responses as some of the healthy controls, reflective of the limits of the amplitude response in firefighters. It is also possible that the results reflect a combined effect of exposures, not entirely limited to noise. As with many occupational groups, firefighters are exposed to a number of toxic agents that may also render the inner ear structures vulnerable (Bolstad-Johnson et al., 2000; Austin et al., 2001; Fechter et al., 2002; Campo et al., 2013). However, our results are consistent with that observed in other known noise-exposed groups with widespread exposures, such as veterans (Akin et al., 2012). Importantly, these findings provide the first evidence of early changes in “young” ears and with limited noise-exposure compared to previously described groups (<1 year mean age, 24 years). The hallmarks of accelerated ageing in the vestibular system and their clinical expression remains to be characterized. A major limitation is the lack of animal models that easily translate to humans. Much of the work in characterizing and understanding the otoliths comes from animal models through VsEP studies, whereas clinical methods rely on the cVEMP response. In the auditory system, we are able to use animal models to explore effects of noise using tests that directly translate to the clinic, such as the otoacoustic emission and the auditory brainstem response (Furman et al., 2013; Fernandez et al., 2015; Kujawa and Liberman, 2015; Bramhall et al., 2018). It would follow that more directly translational animal models will help to further characterize the effects described here toward early diagnosis, prevention and therapeutics. In a companion paper (Raciti et al., 2023), we discuss one such reliable and repeatable cVEMP testing set up and protocol and characterize the evoked responses in a preclinical rodent model. Search for electrophysiological indices of hidden vestibular loss in humans is complicated by these factors. Given that we are identifying a potential hidden vestibular loss while controlling for hearing, we selected to treat ears as individual.

Overall, there is a noise-related decline of vestibular function even in the presence of normal auditory thresholds and functional balance. Studies of the effects of ageing on cVEMP responses indicate that amplitude and latencies changes are limited to older adults, with no observable changes in those less than 50 years of age (Agrawal et al., 2012). Despite participants in our study coming from a relatively younger cohort, the firefighters had measurable changes in vestibular function even within the year of work in the fire service. In the absence of any vestibular symptoms or auditory indicators of NIHL, these early effects on the vestibular system point to a potential hidden vestibular loss. The results are consistent with a noise acceleration of age-related changes in cVEMP. Although, this cannot be fully ascertained by the results herein. Other factors, in particular repeated over exposures, could be the cause behind the cVEMP changes. However longitudinal measurements in the firefighters and matched controls would be needed to further study the effects of contributing factors such as age and co-morbidities. Early detection of noise-induced vestibular loss with cVEMPs has diagnostic promise, although improved translational models are needed in combination with large-scale well-controlled cohort and longitudinal studies to develop methods that are both adequately sensitive and specific to detect the presumably small changes that occur over time with repeated noise overexposure. Additionally, improved translational tools are needed to help address the lack of understanding on how risk varies with contributing factors such as age, ototoxin exposure, and genetics as noise-injury accumulates.

## Data availability statement

The raw data supporting the conclusions of this article will be made available by the authors, without undue reservation.

## Ethics statement

The studies involving humans were approved by Institutional Review Board, University of Miami. The studies were conducted in accordance with the local legislation and institutional requirements. The participants provided their written informed consent to participate in this study.

## Author contributions

HS, LV, and SR contributed to conception and design of the study. HS, LV, BK, and DL-L collected and analyzed the data. BK organized the database. HS performed the statistical analysis and drafted the manuscript. LV and CK wrote sections of the manuscript. All authors contributed to the article and approved the submitted version.
